# DNA Repair Enzyme Poly(ADP-Ribose) Polymerase 1/2 (PARP1/2)-Targeted Nuclear Imaging and Radiotherapy

**DOI:** 10.3390/cancers14051129

**Published:** 2022-02-23

**Authors:** Nghia T. Nguyen, Anna Pacelli, Michael Nader, Susanne Kossatz

**Affiliations:** 1 Department of Nuclear Medicine, University Hospital Klinikum Rechts der Isar and Central Institute for Translational Cancer Research (TranslaTUM), School of Medicine, Technical University Munich, 81675 Munich, Germany; nghia.nguyen@tum.de; 2 Department of Nuclear Medicine, University Hospital Essen, University of Duisburg–Essen, 45147 Essen, Germany; anna.pacelli@uk-essen.de (A.P.); michael.nader@uk-essen.de (M.N.)

**Keywords:** PARP1 inhibitors, DNA repair, PET imaging, radiotherapy, rucaparib, olaparib, clinical translation

## Abstract

**Simple Summary:**

In parallel to the successful clinical implementation of PARP1/2 inhibitors as anti-cancer drugs, which interfere with the DNA repair machinery, these small molecule agents have also gained attention as vehicles for molecular imaging and radiotherapy. In this review article, we summarize the development and preclinical evaluation of radioactively-labelled PARP inhibitors for positron emission tomography (PET) for many applications, such as selecting patients for PARP inhibitor treatment, response prediction or monitoring, and diagnosis of tumors. We report on early clinical studies that show safety and feasibility of PARP-imaging in humans. In addition, we summarize the latest developments in the field of PARP-targeted radiotherapy, where PARP inhibitors are studied as vehicles to deposit highly cytotoxic radioisotopes in close proximity to the DNA of tumor cells. Lastly, we look at synthetic strategies for PARP-targeted imaging and therapy agents that are compatible with large scale production and clinical translation.

**Abstract:**

Since it was discovered that many tumor types are vulnerable to inhibition of the DNA repair machinery, research towards efficient and selective inhibitors has accelerated. Amongst other enzymes, poly(ADP-ribose)-polymerase 1 (PARP1) was identified as a key player in this process, which resulted in the development of selective PARP inhibitors (PARPi) as anti-cancer drugs. Most small molecule PARPi’s exhibit high affinity for both PARP1 and PARP2. PARPi are under clinical investigation for mono- and combination therapy in several cancer types and five PARPi are now clinically approved. In parallel, radiolabeled PARPi have emerged for non-invasive imaging of PARP1 expression. PARP imaging agents have been suggested as companion diagnostics, patient selection, and treatment monitoring tools to improve the outcome of PARPi therapy, but also as stand-alone diagnostics. We give a comprehensive overview over the preclinical development of PARP imaging agents, which are mostly based on the PARPi olaparib, rucaparib, and recently also talazoparib. We also report on the current status of clinical translation, which involves a growing number of early phase trials. Additionally, this work provides an insight into promising approaches of PARP-targeted radiotherapy based on Auger and α-emitting isotopes. Furthermore, the review covers synthetic strategies for PARP-targeted imaging and therapy agents that are compatible with large scale production and clinical translation.

## 1. Introduction to PARP Inhibitors

Due to continuous exposure to DNA-damaging events and the resulting DNA lesions, such as single-strand breaks (SSBs) and double-strand breaks (DSBs), cells maintain their genomic stability through the activation of numerous DNA damage response mechanisms, such as Base Excision Repair (BER), Homologous Recombination (HR), classical and alternative Non-Homologous End Joining (NHEJ), Nucleotide Excision Repair (NER), and Mismatch Repair (MMR) [1,2,3]. In those DNA damage repair pathways, some of the 17-member family of poly(ADP-ribose)polymerase (PARP) proteins play an essential role. PARP1 (in the following referred to as PARP for simplicity), a multifunctional enzyme of 113 kDa, is the best known and most abundantly expressed family member with multiple functions in DNA repair, genomic stability, and cell death [4]. Different factors, such as high rates of genomic instability, mutational burden, and defects in other DNA repair pathways, such as homologous recombination (HR) (e.g., BRCA1/2 mutations), lead to frequent dependence of tumors on PARP1-mediated DNA repair and high PARP1 expression levels [5]. Consequently, inhibition of PARP-mediated DNA repair was discovered to be an efficient approach to selectively kill tumor cells, which resulted in the development of small molecule PARP inhibitors (PARPi) that bind to the NAD^+^ binding pocket of the catalytic domain of PARP1 and prevent poly(ADP)-ribosylation [6,7,8,9,10]. Most PARPi also bind to the lesser expressed close homologue PARP2 with high affinity, therefore they are often named PARP1/2 inhibitors, which we simplify as PARPi. Intense research efforts have produced a high number of highly affine and selective PARPi, which have advanced to clinical evaluation [11]. 

In addition to monotherapy, mostly involving patients with germline or somatic BRCA1/2 or other HR-associated mutations, combination therapies with chemo-, radio- or immunotherapy have been or are currently investigated in clinical studies [11]. Since 2014, four PARPi (olaparib, rucaparib, talazoparib, and veliparip) have been clinically approved by the Food and Drugs Administration (FDA) and/or the European Medicines Agency (EMA) and one PARPi is approved in China (pamiparib). Olaparib received its first approval in 2014 and is now approved as monotherapy for the treatment of certain types of breast, ovarian, fallopian tube, peritoneal, pancreatic, and prostate cancer, as well as in combination with bevacizumab for ovarian cancer [12,13]. Rucaparib has received approval for the treatment of certain prostate and ovarian cancer patients that have failed other therapies [14]. In 2019, niraparib was approved for treatment of recurrent epithelial ovarian, fallopian tube, or primary peritoneal cancers [15], which was recently followed by approval as first line therapy for the same cancers. Talazoparib is currently approved for patients with locally advanced and metastatic breast cancer with germline BRCA1/2 mutations [16]. The high number of ongoing studies will likely result in additional approvals in the future. 

It is now known that the anti-cancer activity of PARPi is mediated by several mechanisms, but to fully understand the resulting in vitro and in vivo efficacy, as well as resistance to PARPi, is an ongoing field of investigation. Initially, catalytic inhibition of the PARP-mediated DNA repair was considered the main mechanism for PARPi-induced cytotoxicity, leading to synthetic lethality in HR deficient tumors. However, catalytic inhibition alone could not fully explain the effects of PARPi therapy, since, e.g., PARPi with similar PARP1/2 affinity exhibits different potency and sensitivity to PARPi and does not always depend on HR status. PARP trapping was identified as an additional mechanism of PARPi, where the dissociation of PARP enzymes from chromatin is prevented by PARPi binding, leading to replication fork stalling and eventually collapse, if not resolved by other DNA repair mechanisms [17]. Subsequently, pronounced differences in trapping potential of known PARPi were discovered [18], which is discussed to explain their differences in in vivo therapeutic efficacy [19]. New studies also add to a better understanding of response biomarkers and resistance mechanisms to PARPi therapy [20,21]. In addition, histone parylation factor 1 (HPF1) was recently identified as an important co-factor in the induction of PARP1/2 mediated DNA repair [22]. HPF1, which forms a joint active site with PARP1 or PARP2, actually also modulates the binding affinity of some PARPi to PARP1, indicating that HPF1 might directly affect PARP inhibition and trapping and, therefore, response to PARPi [23]. Hence, these additional levels of complexity require improved strategies for patient selection for PARPi therapy. 

## 2. Introduction to Radiolabeled PARP Inhibitors

While the importance of PARPi in cancer therapy is steadily growing, identification of responders and non-responders is still challenging. Next to the requirement of PARP1 expression for PARPi sensitivity, a number of resistance mechanisms are known, which can, e.g., circumvent dependence on PARP-mediated DNA repair or directly affect PARPi binding [20,21,24]. Therefore, non-invasive determination of PARP expression and indirect or direct measurement of PARPi binding could be a promising approach for improved patient selection. Radiolabeled probes offer excellent opportunities to measure PARP expression directly and noninvasively in patients via positron emission tomography (PET) or single photon emission tomography (SPECT). PARP imaging agents could be used as companion diagnostics for PARPi therapy, i.e., to select patients and for non-invasive whole-body PARP imaging to predict and/or monitor the response to PARPi therapy. Furthermore, the widespread overexpression of PARP could be leveraged for diagnostic imaging of tumors that are otherwise difficult to image with standard radiotracers, such as [^18^F]FDG, e.g., brain cancer [25] and head and neck cancer [26]. In addition, it was also suggested that PARPi could act as intranuclear delivery vehicles for therapeutic radioisotopes, including α- and Auger-emitters.

**Figure 1:**
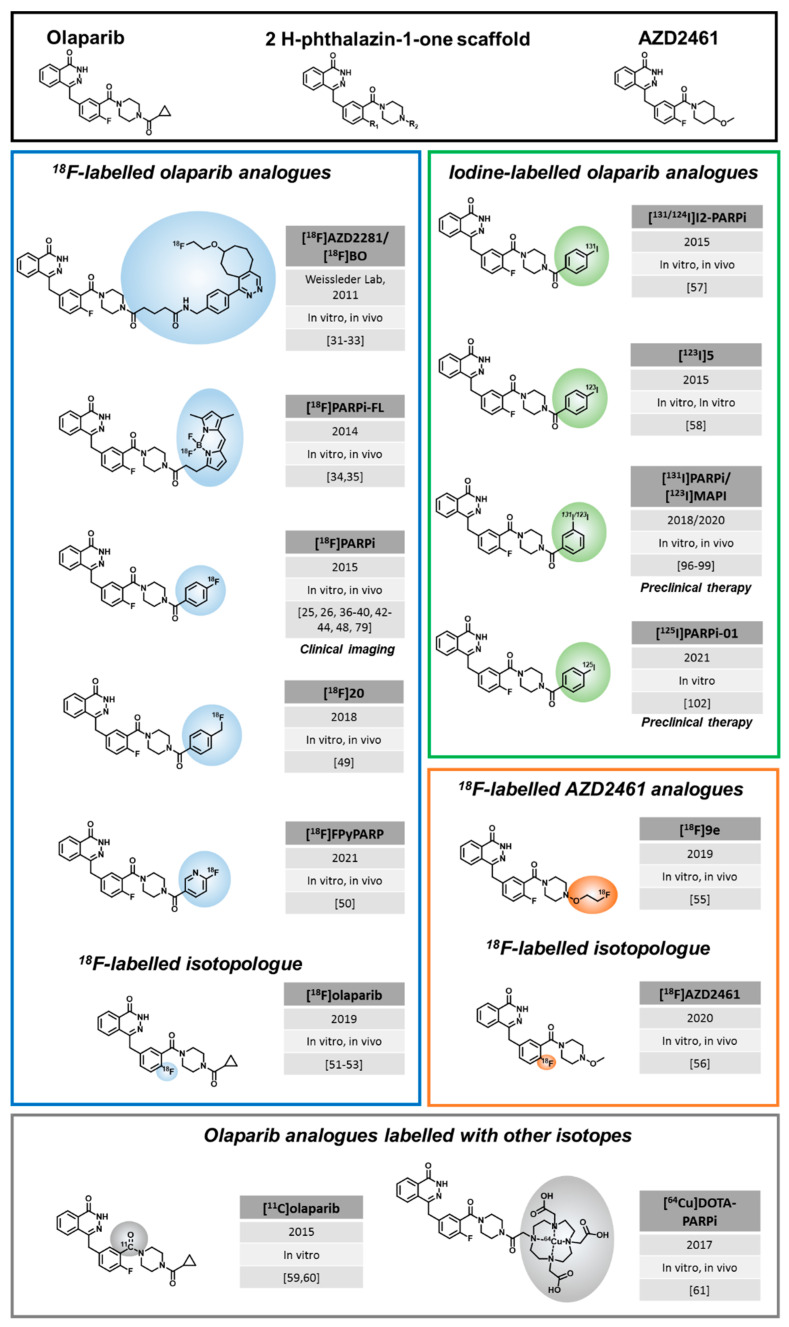
Olaparib-based PARP-targeted imaging and therapy agents. Modifications from the parent PARPi are highlighted by colored circles. Year of first publication, status of development (in vitro, in vivo) and all related publications are mentioned in their order of appearance in the main text. We also pointed out where clinical imaging studies or preclinical therapeutic results are published.

**Figure 2:**
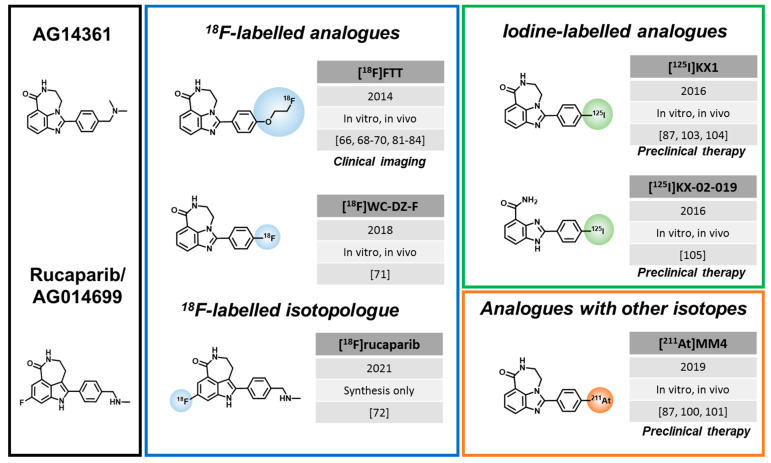
Rucaparib-based PARP-targeted imaging and therapy agents. Modifications from the parent PARPi are highlighted by colored circles. Year of first publication, status of development (in vitro, in vivo), and all related publications are mentioned in their order of appearance in the main text. We also pointed out where clinical imaging studies or preclinical therapeutic results are published.

**Figure 3:**
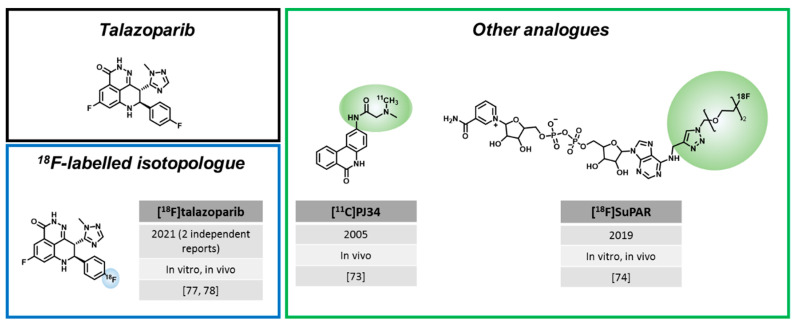
Other PARP-targeted imaging and therapy agents. Modifications from the parent PARPi/parent molecule are highlighted by colored circles. Year of first publication, status of development (in vitro, in vivo) and all related publications are mentioned in their order of appearance in the main text.

To explore these clinically relevant applications, a considerable number of radiolabeled PARPi have emerged, for the most part, in the last ten years. The majority of radiolabeled PARPi are based on the structures of olaparib (Figure 1) and rucaparib (Figure 2). Talazoparib was also recently radiolabeled, as well as a few other PARP-targeting molecules (Figure 3). 

While several comprehensive reviews have previously described preclinical development and translational efforts [25,27,28,29,30], the extensive publication of novel radiolabeled PARPi, as well as clinical study results of the translated PARPi ([^18^F]FTT and [^18^F]PARPi), just in the last three years, warrants a systematic overview of the current status in the rapidly expanding field of PARP imaging and therapy. We included all existing probes in this analysis to gain an understanding of the effects of the different structural modifications compared to the parent PARPi on affinity, selectivity, cell permeability, and pharmacokinetic properties. With the increasing number of clinical studies in mind, we also take a look at the challenges and opportunities regarding synthesis of radiolabeled PARPi in the required quantity and quality for clinical translation. 

## 3. Preclinical Development and Recent Advances in PARP Imaging Agents 

### 3.1. Olaparib-like Radiotracers

The first radiolabeled olaparib analogue emerged in 2011, several years before olaparib’s first clinical approval for the treatment of BRCA-mutated ovarian cancer in 2014 [13]. Several strategies have been explored to attach radiolabels, including ^18^F and iodine isotopes, to the olaparib scaffold, yielding a number of different olaparib-based radiotracers, of whom one, [^18^F]PARPi, has reached clinical evaluation to date. 

The first radiolabeled olaparib derivative was synthesized by Weissleder and colleagues via an inverse-electron Diels–Alder cycloaddition. Accordingly, an ^18^F-labelled trans-cyclooctene (TCO) with a tetrazine-modified olaparib derivative reacted with [^18^F]BO, also called **[^18^F]AZD2281** [31]. [^18^F]BO (IC_50_ = 17.9 ± 1.1. nM) was the first olaparib-based radiotracer to show successful in vivo imaging of breast and ovarian cancer xenografts and its uptake correlated with PARP1 expression [32,33]. However, no further studies were conducted with [^18^F]BO. Subsequently, a bimodal PARP imaging agent, carrying a radiofluorinated fluorescent dye was achieved via Lewis acid-assisted fluoride exchange with triflic anhydride [34]. Although **[^18^F]PARPi-FL** was successfully synthesized and utilized for in vivo imaging of glioblastoma xenografts, it was not further developed due to its rapid metabolic defluorination in vivo [35]. The chemical modifications to achieve [^18^F]BO and [^18^F]PARPi-FL led to a relatively large footprint and bulky structure compared to olaparib, very likely introducing significant changes for target binding and pharmacokinetic properties, but also to the cell penetration ability, which is essential to reach the intranuclear target. Therefore, it is important to note that subsequent radiolabeled olaparib derivatives were structurally more similar and closer in molecular weight to their parent PARPi. 

Replacing the cyclopropyl moiety of olaparib with a fluorobenzene ring led to the radiochemically stable **[^18^F]PARPi**, which was developed in the Reiner lab in 2015. A large body of preclinical work subsequently explored the utility of [^18^F]PARPi for a variety of potential applications, such as diagnosis of brain, head, and neck cancers [26,36,37], quantification of PARPi target engagement [38], efficacy assessment of PARPi treatment, and for differentiation between malignant and non-malignant lesions in lymphomas and gliomas [39,40]. This tracer has also been clinically translated, which is detailed in Section 4. In vitro experiments showed a similar affinity and selectivity profile of [^18,19^F]PARPi and olaparib (IC_50_ value of [^19^F]PARPi: 2.83 nM, olaparib: 5 nM) [37,38], supporting that loss of the cyclopropyl moiety did not affect these properties, aligning with previous reports stating that it mainly increased oral bioavailability [7,41]. Recently, Wilson et al. suggested a simplified and faster two-step, one-pot radiosynthesis with a radiochemical yield of up to 9.6%, compared to the originally published multistep, multi-pot procedure to potentially facilitate production for clinical studies [42].

[^18^F]PARPi was initially evaluated for glioblastoma imaging. In subcutaneous and orthotopic U251 MG xenograft models, the authors found a tumor uptake of 1.8–2.2% ID/g with high specificity (>85% blockable), but only a very low brain uptake, resulting in a tumor-to-brain ratio of 55, providing high signal to noise contrast [37]. [^18^F]PARPi was also able to clearly delineate gliomas with PET imaging in a genetically engineered mouse model, with higher accuracy than [^11^C]Choline and [^18^F]Fluorothymidine. The uptake correlated with PARP1 expression and was validated to be tumor specific in blocking experiments, autoradiography, and by using a fluorescent analogue (PARPi-FL) for microscopic evaluation [25]. The ability of [^18^F]PARPi to penetrate into areas of the brain inaccessible to high molecular weight FITC-Dextran in the genetically engineered glioma mouse model suggests blood-brain barrier (BBB) penetration by the tracer [43], which could enable tumor imaging even if the BBB is not compromised by the tumor. 

Head and neck cancer imaging was identified as another potential application for [^18^F]PARPi. These investigations followed studies showing PARP1 overexpression in oral and oropharyngeal cancer [44,45]. The group previously showed feasibility of diagnostic and intraoperative oral cancer imaging using the fluorescent PARP imaging agent PARPi-FL preclinically and clinically [44,45,46,47]. In a recent study, [^18^F]PARPi PET imaging was evaluated in orthotopic oral cancer models in comparison to [^18^F]FDG [26]. Clinically, FDG-PET is used to determine disease extent and post-therapy surveillance, which is complicated by physiological uptake in the head and neck region. The study showed that [^18^F]PARPi uptake was limited to tumor tissue and showed higher uptake in orthotopic tongue tumor xenografts compared to the surrounding tongue, which was not the case for [^18^F]FDG, indicating its feasibility for clinical applications in head and neck cancer imaging. Another study found that human papilloma virus (HPV)-positive and negative oropharyngeal cancer cells showed similar PARP1 expression and [^18^F]PARPi uptake, suggesting the tracer as an HPV-independent imaging tool for imaging in oropharyngeal cancer patients [36]. 

PARPi are also in clinical studies as mono- and combination treatments in small cell lung cancer (SCLC). In this context, [^18^F]PARPi imaging was introduced as a tool to measure the extent and duration of target engagement of the PARPi’s olaparib and talazoparib in patient-derived xenograft models of SCLC [38]. Since complete inhibition of PARP-mediated DNA repair is essential for efficient therapy, this real-time, non-invasive monitoring approach could be used to optimize dosing and timing of PARPi therapy [38]. A follow-up study later actually showed that treatment of SCLC PDX bearing mice with an efficacious and sub-efficacious dose of talazoparib (0.3 mg/kg vs. 0.1 mg/kg) correlated with [^18^F]PARPi uptake on PET imaging and treatment outcome [48]. 

[^18^F]PARPi was also evaluated as an alternative for [^18^F]FDG for diffuse large B-cell lymphoma (DLBCL) imaging in a syngeneic mouse model [39]. DLBCL treatment can induce inflammation, and [^18^F]FDG PET often faces difficulties in differentiating malignant from inflamed masses, e.g., in lymph nodes [39]. [^18^F]PARPi uptake was significantly higher in DLBCL lymph nodes compared to inflamed and normal lymph nodes, which also reflected PARP1 expression, while [^18^F]FDG uptake was similar in DLBCL and inflamed lymph nodes [39]. 

Lastly, [^18^F]PARPi was tested for its ability to distinguish recurrent tumor from radiation injury [40]. The inability to confidently distinguish these entities is an important clinical problem, e.g., in brain tumors, because it can result in delayed treatment decisions. In a mouse model of experimental radiation necrosis, [^18^F]PARPi showed no avidity to radiation injury (lesion/contralateral ratio: 1.02), while the brain tumor imaging tracer [^18^F]Fluorethyltyrosine showed increased lesion uptake (lesion/contralateral ratio: 2.12), indicating [^18^F]PARPi could be a more specific marker to discriminate these two pathologies non-invasively [40]. 

The Pimlott lab introduced **[^18^F]20** as a PET imaging agent for glioblastoma in 2018. This tracer has a methylfluorobenzene instead of the cyclopropyl moiety of olaparib and is thus structurally very similar to [^18^F]PARPi with its fluorobenzene. Although it had a low IC_50_ value (1.3 nM in G7 human glioblastoma cells) and favorable tumor uptake (3.6% ID/g after 120 min), [^18^F]20 was eliminated from further investigations due to observed high skeletal uptake (8.5% ID/g) due to defluorination [49]. 

Both clinically translated PET imaging agents, [^18^F]FTT and [^18^F]PARPi, are characterized by hepatobiliary clearance, which complicates imaging of abdominal lesions, e.g., liver metastases. To address this limitation, Stotz et al. introduced **[^18^F]FPyPARP** as a less lipophilic variant by exchanging the fluorobenzoyl residue with a fluoronicotinoyl group [50]. A side-by-side in vivo comparison of [^18^F]FPyPARP to [^18^F]FTT and [^18^F]PARPi revealed a partial shift to renal clearance, but since tumor-to-liver ratios remained well below “1”, it is likely that further modifications and a stronger shift to renal clearance would be required for PARP1 imaging of abdominal lesions. 

In 2019, Wilson et al. (Cornelissen lab) reported **[^18^F]olaparib**, which is an isotopologue of olaparib, and hence the first directly radiolabeled PARPi without structural modifications [51]. Radiolabeling was achieved via a copper-mediated ^18^F-fluorodeboronation of a protected boronic pinacol ester precursor in a synthesis time of 135 min and an activity yield of 18% [51]. In vitro studies showed a correlation of [^18^F]olaparib uptake with PARP1 expression levels in pancreatic ductal adenocarcinoma (PDAC) cells. PET studies in mice bearing PSN-1 xenografts revealed a tumor specific uptake of 3.2% ID/g, which could be blocked. Furthermore, [^18^F]olaparib uptake increased by 70% after irradiation with 10 Gy, corresponding to an increase PARP1 expression in tumors [51]. Meanwhile, Guibbal et al. established procedures for automated radiosynthesis of [^18^F]olaparib (120 min, activity yield: 6%), which are compatible with Eckert and Ziegler Modular Lab systems, offering promising perspectives for production for clinical studies and routine use [52]. Furthermore, Bowden et al. was able to introduce a feasible automated copper-mediated radiofluorination, which led to an increase in activity yield (41%) and radiochemical yield (80%) [53]. Additional clinical and preclinical data obtained with [^18^F]olaparib are eagerly awaited by the scientific community.

Since treatment resistance to olaparib is often associated with rapid elimination via drug transporters, especially p-glycoprotein (P-gp), AZD2461 was developed in 2016 as the next generation PARPi. AZD2461 showed similar anticancer potency as olaparib in vitro and in vivo but is a poor substrate for drug transporters. In addition, it showed lower levels of haematological toxicity in mice and was found to be a weaker inhibitor of PARP3 than olaparib [54]. Evading P-gp drug transport should also lead to better penetration of the BBB. To test this hypothesis and investigate the role of PARP1 in neuroinflammation and neurodegenerative diseases, Reilly et al. (Mach lab) developed an ^18^F labelled analogue of AZD2461 in 2019, called **[^18^F]9e.** However, [^18^F]9e showed non-appreciable brain-uptake in non-human primates, suggesting that [^18^F]9e does not cross the BBB and is hence not suitable to investigate PARP1 in neurodegenerative diseases [55]. 

Almost in parallel, a radiofluorinated isotopologue of AZD2461 was synthesized via copper-mediated ^18^F-fluorodeboronation (Gouverneur and Cornelissen lab). **[^18^F]AZD2461** was evaluated in pancreatic cancer cell lines and a xenograft mouse model of pancreatic cancer in comparison to [^18^F]olaparib, which was developed in the same lab [56]. Cellular uptake of [^18^F]AZD2461 in PSN-1 cells was less than 50% compared to the [^18^F]olaparib. Interestingly, blocking with olaparib or AZD2461 only reduced the [^18^F]AZD2461 uptake to 70% and 25% of the initial binding, respectively, while both olaparib and AZD2461, could completely block [^18^F]olaparib uptake. In vivo, [^18^F]AZD2461 uptake could also not be blocked completely, but curiously olaparib was more efficient at blocking than AZD2461 [56]. These results could suggest that AZD2461 has other, currently unknown, targets in addition to PARP1 and PARP2 and is hence less suitable as a PARP imaging agent. 

In parallel to ^18^F-labelled olaparib analogues, iodinated derivatives based on the same 2H-phthalazin-1-one scaffold of [^18^F]PARPi were developed, since the variety of iodine isotopes could enable imaging with PET (e.g., ^124^I and SPECT (e.g., ^131^I), but also radionuclide therapy (e.g., ^131^I, ^123^I and ^125^I). Here, it needs to be considered that the large molecular weight of iodine could negatively affect the molecule’s membrane penetration capability and pharmacokinetics and hence, tumor uptake. In 2015, Salinas et al. synthesized a series of *meta* and *para*-iodinated olaparib analogues with different linker lengths between the aromatic ring and the olaparib core, resulting in compounds with IC_50_-values between 9 and 107 nM. The group identified **[^124/131^I]I2-PARPi** (*para*-iodinated) as the lead candidate, which showed high PARP1 affinity (IC_50_ = 9 nM) and specificity, shown by blocking. In vivo, [^124/131^I]I2-PARPi was able to delineate orthotopic glioblastoma xenografts using PET as well as SPECT imaging and yielded tumor-to-brain ratios of 40 ± 6.3 in U251 MG xenografts 2 h p.i. [57]. 

Simultaneously, Zmuda et al. reported in 2015 an ^123^I-labelled version of I2-PARPi using the same precursor and coupling conditions as Salinas et al., called **[^123^I]5** [58]. This radiotracer was evaluated as a potential SPECT imaging agent for glioblastoma as well and reached tumor-to-muscle ratios of 5.6 ± 2 at 2 h p.i. in a subcutaneous U87 MG model [58].

One group also evaluated ^11^C as possible radionuclide to create **[^11^C]olaparib**. However, the work of Andersen et al. showed that the palladium complexes which were used as a precursor for the labeling reaction were unstable [59]. Despite a continuing effort to develop optimized reaction conditions for the ^11^C-labeling reaction [59,60], fast progress with ^18^F-labeling and its longer half-life led to a stronger focus on ^18^F-labelled PARP inhibitors. 

Reporting about “exotic” radionuclides, the work of Huang et al. needs to be mentioned. Therefore, **[^64^Cu]DOTA-PARPi** with a ^64^Cu-chelating system was conjugated to the olaparib precursor. Unfortunately, although tumor uptake in mesothelioma mice models reached 3.45% ID/g after 1 h, the conjugation of the DOTA-chelating moiety led to a decrease in binding affinity by 40 [61]. These findings underline that such large structural modifications compared to the parent PARPi cannot be tolerated in the design of PARP imaging agents. 

### 3.2. Rucaparib-like Radiotracers

The rucaparib scaffold was developed in 2008 by a collaboration between the University of Newcastle and Agouron Pharmaceuticals [62]. Menear et al. followed up the development and discovered the inhibitory potential of rucaparib towards PARP [41], which led its first phase I clinical study in combination with temozolomide in patients with advanced, solid tumors [63]. Ten years later, in 2018, the EMA approved rucaparib to be used in patients with HR deficient ovarian cancer [64]. Now, it is also FDA-approved for the treatment of HR deficient metastatic castration-resistant prostate cancer [65]. In parallel, several radiolabeled rucaparib analogues were developed. 

Zhou et al. (Mach lab) developed the first ^18^F-labelled radiotracer structurally closely related to rucaparib, [^18^F]F12, later called **[^18^F]Fluorthanatrace**, in 2014 [66]. [^18^F]Fluorthanatrace (short: [^18^F]FTT) was derived from AG14361 [67], not AG014699/rucaparib, by replacing the dimethyl phenylmethanamine with ^18^F-fluoroethoxy benzene [62]. AG14361 was a former candidate for clinical development by Agouron, but later rucaparib was chosen due to better in vitro potency and in vivo efficacy [62,63]. Structurally, rucaparib features a fluorination and possesses an amine group on the indole moiety, which are absent in AG14361. [^18^F]FTT displayed a good affinity (IC_50_ = 6.3 nM) towards PARP1 and showed specific tumor uptake (3–5% ID/g 1 h p.i.) in MDA-MB-231 and MDA-MB-436 xenograft models [66]. In a panel of breast cancer cell lines, [^18^F]FTT uptake was compared in BRCA-mutant HCC1937 (high PARP1 expression) to the BRCA-wildtype MDA-MB-231 and MCF-7 cells and corresponded to these different expression levels [68]. In vivo imaging showed the best tumor delineation in the HCC1937 xenografts, with tumor-to-muscle ratios of 1.9 [68]. Another study from the same lab also found higher [^18^F]FTT uptake in BRCA-mutant (SNU-251) than BRCA-WT (SCOV3) cells, corresponding to protein expression levels. Since expression and radiotracer uptake was higher in the BRCA-mutant cell line, corresponding to its higher sensitivity to PARPi treatment and radiation, the authors suggested that [^18^F]FTT could be used to predict treatment efficacy [69]. The same approach was investigated in a study focused on ovarian cancer. Here, it was first shown that PARP1 knockout cells and mice showed resistance to PARPi treatment, confirming that PARP1 expression is a requirement for PARPi sensitivity. The authors observed that [^18^F]FTT tumor uptake was decreased in olaparib treated animals compared to untreated animals, concluding that the tracer is suitable to measure PARP1 expression in vivo [70].

In 2018, Zhou et al. developed a modified version of [^18^F]FTT, called **[^18^F]WC-DZ-F** [71]. The radiotracer was radio-fluorinated directly at the *para* position of the benzene ring, in exchange for the fluoroethoxy group of [^18^F]FTT. This compound was characterized in a subcutaneous prostate cancer model, where tumor uptake was around 4% ID/g 2h p.i. [71]. Although [^18^F]WC-DZ-F showed a higher in vivo blood stability compared to [^18^F]FTT (78.5% vs. 13% at 30 min), substantial nonspecific uptake in bone and muscle were observed in the biodistribution data, limiting the potential advantages over [^18^F]FTT [71].

Recently, the first ^18^F-radioisotopologue of rucaparib was developed (Cornelisson and Gouverneur labs) using a synthesis strategy involving Cu(II)-mediated ^18^F-fluorodeboronation followed by reductive amination, to obtain **[^18^F]rucaparib** where the fluorination took place at the aromatic ring system of the benzimidazole core [72]. Similar to olaparib/[^18^F]olaparib, [^18^F]rucaparib is expected to have identical properties and pharmacokinetics as its parent molecule. The first in vivo imaging data with [^18^F]rucaparib are eagerly awaited. 

### 3.3. Radiotracers Based on Other PARPi

In addition to the extensive research efforts with regards to olaparib and rucaparib-like radiotracers, a few PARPi based on other natural structures have been developed. 

In 2005, before the discovery of rucaparib and olaparib as PARPi, Tu et al. were working on the very first example of a PET tracer targeting PARP [73]. **[^11^C]PJ34** was a phenanthridinone derivative, which was able to block NAD^+^ from its natural binding site on the PARP enzyme. Importantly, hyperactivation of PARP leads to the depletion of NAD^+^ inside the cells, which can induce necrosis or lead to related diseases, such as diabetes [73]. Using streptozotocin-treated rats (type I diabetes model), a high uptake of [^11^C]PJ34 in the target organs, the liver and pancreas, was observed. This indicated the possibility of [^11^C]PJ34 to target PARP during its hyperactivation, which is a key driving mechanism for necrosis-related diseases [73]. However, further studies with this radiotracer were not conducted. 

While all other PARP imaging approaches are based on radiolabeled PARPi, Shuhendler et al. pursued a different approach and developed a radiofluorinated NAD^+^ analogue with the goal to image parylation activity instead of PARP expression [74], since PARP activity could be a better predictor for PARPi therapy response. Indeed, **[^18^F]SuPAR** showed increased tumor uptake in HeLa and MDA-MB-231 xenografts after radiation treatment, which significantly correlated with increased PAR levels after the DNA damage inducing treatment. The specificity of [^18^F]SuPAR was shown by a decreased tumor uptake after blocking with the PARPi talazoparib in mice. Despite these promising results, it should be noted that in vivo assessment of PARP activity was complicated by the fact that NAD^+^ also serves as substrate for other enzymes and plays important roles in enzyme catalyzing redox reactions.

In addition, the first talazoparib-based radiotracers have recently emerged. Talazoparib (IC_50_ = 0.6 nM) is a PARPi with a similar affinity (IC_50_ = 0.6 nM), but much higher potency than olaparib and rucaparib (IC_50_ = 1.9 nM and 2.0 nM, respectively), which is often attributed to its high PARP-trapping capacity and its broader selectivity profile [18,75]. Of note, talazoparib is given clinically at much lower daily doses (1 mg/day) than olaparib and rucaparib (300 mg twice daily), due to its higher potency and toxicity. Talazoparib was approved by the FDA (2018) and EMA (2019) for the treatment of germline BRCA-mutated, HER2-negative metastatic breast cancer [16]. It is further clinically tested, e.g., in metastatic breast cancer patients with a deleterious somatic BRCA mutation and in men with DNA repair defects additional to their metastatic castration-resistant prostate cancer. Two research groups reported the radiosynthesis of **[^18^F]talazoparib** isotopologues in 2021 using different strategies. Zhou et al. (Katzenellenbogen and Xu lab) largely followed procedures in line with the original non-radioactive synthesis of talazoparib [76] and pursued early stage ^18^F incorporation [77], while Bowden et al. (Maurer lab) established a late stage ^18^F incorporation route to obtain the radiotracer [78]. Bowden et al. achieved automated radiosynthesis of [^18^F]talazoparib, yielding an enantiomerically pure compound. This is important, since talazoparib possesses two distinct chiral centers, of which only the (*8S*, *9R*)-diastereomer is a potent PARPi [78]. Subsequent in vitro experiments showed a blockable radiotracer uptake of ~22% of added activity in HCC1937 cells compared to the less potent (*8R*, *9S*)-diastereomer with ~0.3% uptake in the same cell line [78]. In vivo biodistribution data in HCC1937 xenograft-bearing mice showed a tumor uptake of 3.7 ± 0.7% ID/g, but tumor-to-muscle ratios of only 1.8 ± 0.4 at 2.5 h p.i. [78]. Zhou et al. (Katzenellenbogen and Xu lab) also synthesized [^18^F]talazoparib with high chiral purity in an alternative synthetic route, involving less steps and different fluorination conditions compared to [78]. [^18^F]talazoparib showed high tumor uptake in in PC-3 prostate cancer xenografts, which slightly increased from 4 h (3.8 ± 0.6% ID/g) to 8 h p.i. (4.5 ± 0.3% ID/g) [77]. The biodistribution was rather similar to Bowden et al. and showed high uptake in liver, spleen, kidney, and pancreas that only slightly reduced over time. Imaging data are not reported in this study. 

Both studies indicate that [^18^F]talazoparib shows slower washout from organs than olaparib and rucaparib-based radiotracers, with high organ uptake in the spleen, liver, and kidneys, which could be challenging for imaging applications with ^18^F. Nevertheless, Zhou et al. suggest that the prolonged tumor retention could be an advantage for radiotherapy applications, which could be studied using bromo- and iodo-derivates reported in the same publication [77]. 

## 4. Clinical Evaluation of PARP Imaging Agents

### 4.1. [^18^F]PARPi

In addition to the large body of preclinical work, which investigated a variety of potential clinical scenarios, results from two clinical studies centered on [^18^F]PARPi PET imaging were published to date, both in 2021 (Table 1). The first-in-human trial of [^18^F]PARPi investigated safety and feasibility of PET/CT imaging in head and neck cancer patients (NCT03631017) [79]. PET/CT scans and analysis of blood samples of 11 patients with oral and oropharyngeal cancer were obtained 30, 60, and 120 min post injection. The patients received an [^18^F]FDG scan as well, which was compared to [^18^F]PARPi. The tracer was well tolerated by all patients with only one patient experiencing grade 1 mucositis. All primary tumors (n = 10) and FDG-avid lymph nodes (n = 34) could be visualized with [^18^F]PARPi with an average SUV_max_ of 2.8 ± 1.2 at the 120 min timepoint, which yielded the highest lesion-to-background contrast. Rapid clearance of [^18^F]PARPi from healthy organs was observed between the 30 and 120 min timepoints, whereas the activity persisted longer in primary tumors and the metastatic lymph nodes [79]. The study reports that [^18^F]FDG uptake yielded, on average, higher SUV_max_ values in tumors and metastatic lymph nodes, but [^18^F]PARPi uptake was less variable. Furthermore, the authors report that [^18^F]PARPi imaging resulted in an average dose of 3.9–5.2 mSv per scan, which is lower than an average FDG scan (8.1 ± 1.2 mSv) [80]. Interestingly, on top of all FDG-avid lesions, [^18^F]PARPi showed uptake in six additional lymph nodes. However, the phase I study protocol did not allow the biopsy of these lesions or conduct general histological confirmation of the imaging results. In this study, patients received on average 290 pmol [^18^F]PARPi, which is 6.7 orders of magnitude lower than a typical daily dose of olaparib (2 × 300 mg) during an active treatment cycle [79]. 

**Figure 4:**
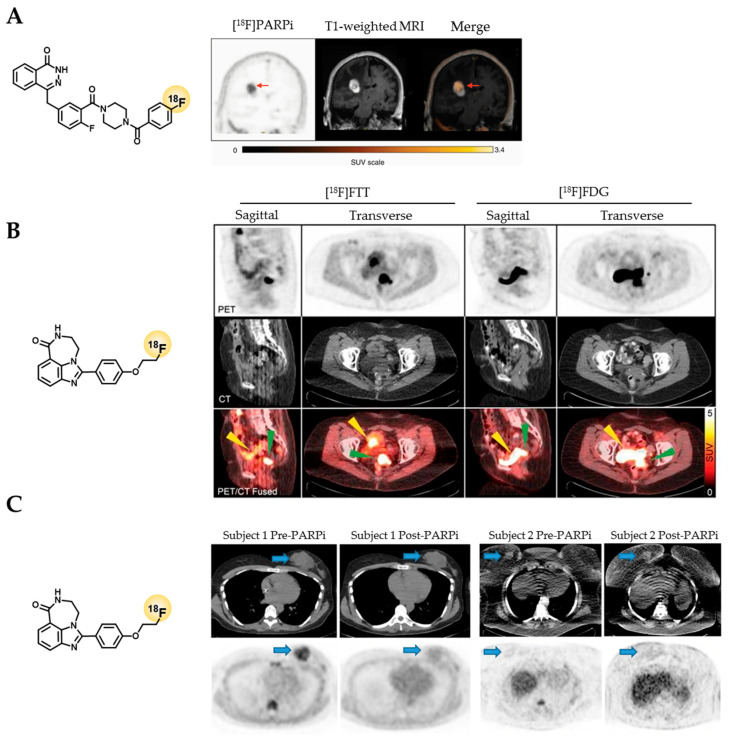
Selected clinical PET imaging results of [^18^F]PARPi and [^18^F]FTT. (**A**) PET/MR imaging of [^18^F]PARPi (NCT04173104) in a brain cancer patient showed heterogenous uptake, which corresponded to areas of higher and lower PARP1 expression in histological analysis [43]. (**B**) [^18^F]FTT imaging of an ovarian cancer patient (NCT02637934) showed clear tumor visualization (green arrow) and delineation (SUV_max_ = 5 g/mL) and absence of bladder uptake observed with [^18^F]FDG PET (yellow arrow) [70]. (**C**) [^18^F]FTT uptake in breast cancer patients (NCT03846167). Subject 1 had clear tumor uptake pretherapy (SUV_max_ breast 4.7 g/mL) and a blockade of uptake posttherapy (SUV_max_ breast 2.4 g/mL) and went on to have a response to PARPi. Subject 2 had minimal uptake pretherapy (SUV_max_ breast 2.3 g/mL) and a similar uptake posttherapy (SUV_max_ breast 2.4 g/mL) and had progression on PARPi [81]. Copyright notice: (A) Reprinted with permission from [43], Copyright, 2021, Society of Neuro-Oncology. (**B**) Reprinted with permission from [70], Copyright, 2018, American Society for Clinical Investigation. (**C**) Reprinted from [81] under Creative Commons CC BY 4.0.

In the second clinical study of [^18^F]PARPi, which was focused on brain cancer (NCT04173104), PET/MR imaging of five brain cancer patients was conducted [43]. The tracer showed higher uptake in active brain tumor lesions (SUV_mean_ = 1.16) compared to regions associated with treatment-related changes (SUV_mean_ = 0.45) at 60 min p.i. and tracer uptake could be correlated with PARP1 expression via immunohistochemistry [43]. Although only an anecdotal observation, heterogeneity in intratumoral [^18^F]PARPi uptake in one patient could be connected to areas of high and low PARP1 expression in histological analysis (Figure 4A). Overall, the study indicates uptake specificity, the ability to cross the BBB, and confirms the very low uptake of [^18^F]PARPi in normal brain tissue, which is promising for brain cancer imaging, but larger patient cohorts are needed to confirm these results.

**Table 1:** Overview of all currently ongoing or finished clinical trials of introduced radiotracers.

Tracer	Conditions	Planned/Final Cohort Size	Status (Clinicaltrials.Org)	NCT Number	Study Parameters	Results Published
[^18^F]PARPi	Head and neck cancer	12	Finished ^1^	NCT03631017	Static [^18^F]PARPi and [^18^F]FDG PET	[79]
	New or recurrent brain tumors	8	Ongoing ^1^	NCT04173104	Static [^18^F]PARPi and [^18^F]FDG PET	[43]
[^18^F]FTT	Head and neck, lung, ovarian, gastric, or pancreatic cancer	50/16	Finished ^2^	NCT02469129	Static [^18^F]FTT PET	[82,83]
	Epithelial ovarian, fallopian tube, or primary peritoneal cancer	30/20	Ongoing ^3^	NCT02637934	Dynamic and static [^18^F]FTT and [^18^F]FDG PET, IF/IHC correlation	[70,84]
	Primary breast cancer	30/30	Finished ^3^	NCT03083288	Static [^18^F]FTT PET	[85]
	Primary or metastatic breast cancer	30/4	Ongoing ^4^	NCT03846167	[^18^F]FTT PET pre and post PARPi therapy	[81]
	Prostate cancer	30	Finished ^3^	NCT03334500		/
	Pancreatic cancer	30	Ongoing ^3^	NCT03492164		/
	Solid tumors	120	Ongoing ^5^	NCT03604315		/
	Glioblastoma	12	Ongoing ^4^	NCT04221061		/
	Breast Cancer	36	Not yet recruiting ^6^	NCT05226663		/

^1^ At Memorial Sloan Kettering Cancer Center in New York, United States. ^2^ At Washington University School of Medicine in Missouri, United States. ^3^ At Abramson Cancer Center of the University of Pennsylvania in Pennsylvania, United States. ^4^ At University of Pennsylvania in Pennsylvania, United States. ^5^ At National Cancer Institute in Texas, United States. ^6^ At MD Anderson Cancer Center, Houston, Texas, United States.

### 4.2. [^18^F]FluorThanatrace

[^18^F]FTT is the PARP imaging agent with the most extensive clinical evaluation to date. At the time of this writing, nine studies are registered in clinicaltrials.gov (keyword: FluorThanatrace; accessed 11 February 2022). Four of them are early phase 1 (phase 0), three are phase 1 trials, one was not assigned a phase, and one is phase 2. The first clinical data, published in 2017 (NCT02469129), included a small cohort of eight patients with different malignancies and provided the first evidence that clinical [^18^F]FTT imaging is feasible [82]. PET images from this study showed visible [^18^F]FTT uptake in tumor regions from one out five patients with measurable tumors who had a biphenotypic hepatocellular carcinoma/cholangiocarcinoma [82]. An erratum to the study corrected that a patient with pancreatic ductal adenocarcinoma, who was originally reported to show [^18^F]FTT uptake, did not demonstrate [^18^F]FTT uptake above the background activity [83]. The effective dose was estimated at 6.9 mSv, which is in a similar range of a [^18^F]FDG PET scan.

In 2018, Makvandi et al. reported results from NCT02637934, where 18 patients with epithelial ovarian cancer types underwent [^18^F]FTT and [^18^F]FDG PET/CT imaging (Figure 4B) [70]. Researchers observed [^18^F]FTT uptake in patients using PET/CT with maximum standardized uptake values (SUV_max_) ranging from 2–12 g/mL (clear delineation of tumor region for SUV_max_ > 5 g/mL). Further correlation of PET imaging with PARP1 immunofluorescence staining and autoradiography was found, but not with [^18^F]FDG imaging [70]. Recently, more data from this trial were published, with a special focus on the pharmacokinetics of [^18^F]FTT [84]. Data from 14 patients over the course of 60, 90, and 180 min post-injection, were analyzed. For the 0–60 min dynamic scan time points, the kinetic parameters (e.g., 2-tissue-compartment model with reversible binding) and SUV_max_ values were in correlation with PARP immunofluorescence data (r = 0.80 and r = 0.93, respectively). Stability of the radiotracer after 60 min was confirmed via computational kinetic analysis showing 59% of parent [^18^F]FTT was still intact in pooled plasma samples. Interestingly, at longer dynamic scan times of t = 110 min and 199 min, the tumor uptake increased, suggesting a possible irreversible binding (model) as a consequence of PARP trapping [84]. 

Published results are also available from NCT03083288 [85] and NCT03846167 [81]. Both studies evaluated [^18^F]FTT for the quantification of PARP expression levels in breast cancer patients using PET/CT imaging. In NCT03083288, 30 breast cancer patients (stage I to IV) with a range of breast cancer phenotypes (estrogen receptor-positive, human epidermal growth factor receptor-positive or triple negative) were enrolled and the BRCA status was analyzed. The study showed that [^18^F]FTT uptake was highly variable among the different subtypes of breast cancer and showed similar variability within each subtype (SUV_max_ = 2.6–11.3 g/mL). Furthermore, patients with and without BRCA1/2 mutations had a similar range of tumor uptake levels (SUV_max_ = 2.9–11.3 g/mL) [85]. This is interesting, since BRCA1/2 status is currently used as only a biomarker for PARPi treatment eligibility, but the response patterns are still not well understood. Potentially, varying levels of PARP expression could also contribute to the treatment response within the eligible patient population. Clinical studies involving pre-treatment PARP-PET and correlating the uptake to the treatment response would be required to answer this question. In NCT03846167, four breast cancer patients with invasive ductal carcinoma (stage III/IV, 3 triple-negative and 1 estrogen receptor-positive) underwent [^18^F]FTT PET/CT imaging pre- and one week post-PARPi treatment (Figure 4C) [81]. Within this group, three patients had moderate [^18^F]FTT uptake pre-treatment (SUV_max_ range: 4.2–6.8 g/mL) and subsequently showed stable disease or tumor regression in response to PARPi treatment. The fourth patient did not show [^18^F]FTT uptake above background in any lesion pre-treatment and also did not respond to PARPi therapy. The study also found that [^18^F]FTT uptake was reduced to background levels in all patients in the “post treatment” scan [81]. Although not clearly stated, we assume that PARPi treatment was still ongoing at the time of the second scan in order to show PARPi target engagement. Despite the small number of patients, these results are promising and warrant further studies into prediction of treatment response and measurement of target engagement in clinical studies. 

## 5. Current Status of PARP-Targeted Radiotherapy

PARP-targeted radiotherapy offers the exciting prospect of delivering cytotoxic radiation directly to the tumor cell nucleus, and therefore, the DNA, instead of the cell membrane or tumor microenvironment, raising hopes to more efficiently introduce DNA damage compared to extranuclear radioligand therapy agents. Especially the use of radioisotopes with strong linear energy transfer (LET) and short path lengths, i.e., Auger electron emitters like ^123^I (t_1/2_ = 13.2 h [86]) or ^125^I (t_1/2_ = 60 d [87]) and α-emitters, such as ^211^At (t_1/2_ = 7.2 h [88]) could find a highly effective application using PARPi as intranuclear delivery vehicles [89,90]. 

Auger electrons have a high LET of 4–26 keV/µm with an extremely short tissue range of 2–500 nm, which means that they only cause lethal damage when emitted in direct vicinity to sensitive structures, such as the DNA or the cell membrane [89,91]. A-emitters have an even higher LET of about 80 keV/µm and a moderate pathlength of 50–100 µm, which covers up to 5 cell diameters. Some studies have reports that only 1–10 α-particle traversals are necessary to effectively kill a target cell [92]. Β-emitters, such as ^131^I or ^177^Lu, have a greater path length of up to 1–12 mm with an LET of 0.2 keV/µm, meaning they can be suitable to treat larger tumor masses but can also cause damage in adjacent tissues and organs [89]. Key characteristics of the therapeutic radioisotopes that have been conjugated to PARPi to date are summarized in Table 2 and main parameters and outcomes from preclinical in vivo studies are summarized in Table 3. 

### 5.1. Olaparib-like Radiotherapeutics

**Figure 5:**
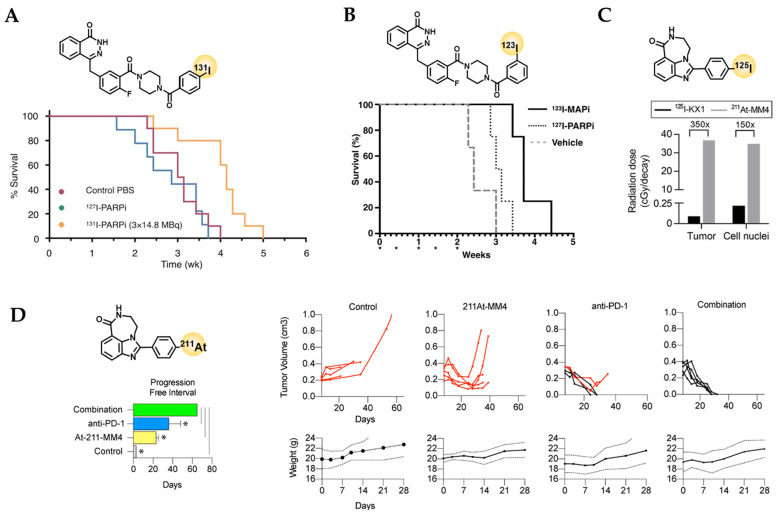
In vivo evaluation of therapeutic efficacy of PARP-targeted radioligands using therapeutic radioisotopes. See Table 3 for corresponding survival data. (**A**) Treatment of subcutaneous p53-deficient U87 glioblastoma with 3 × 14.8 MBq intratumoral dose of [^131^I]PARPi [96]. (**B**) Therapeutic efficacy of 5 × 74 MBq [^123^I]MAPi i.v. in subcutaneous p53-deficient HCT116 colorectal cancers compared to [^127^I]MAPi and vehicle [99]. Stars represent days of treatment. (**C**) In vivo tumor radiation dosimetry modelling revealed a 150 times higher tumor-nucleus dose per decay of the α-emitter [^211^At]MM4 compared to the Auger-emitter [^125^I]KX1 [87]. (**D**) Treatment of syngeneic glioblastoma (GL26) with a single i.v. dose of ~720 kBq [^211^At]MM4 and in combination with anti-PD-1 checkpoint inhibitor [100] Stars indicate signifant differences (Anova, *, *p* < 0.05). *Copyright notice:* (A) Reprinted with permission from [96]. Copyright 2018, SNMMI. (**B**) Reprinted with permission [99]. Copyright 2021, American Chemical Society. (**C**) Reprinted with permission from [87]. Copyright 2020, SNMMI. (**D**) Reproduced with permission from [100]. Copyright 2021, American Chemical Society.

The therapeutic efficacy of the β-emitting radiotracer **[^131^I]PARPi** ([^131^I]I1-PARPi from [57]), was evaluated in mice bearing U251 MG or U87-p53 human glioblastoma xenografts [96]. Specific uptake and tumor retention after intratumoral injection of the tracer was shown in two ways–the tracer could block uptake of [^18^F]PARPi and uptake of [^131^I]PARPi could be blocked by olaparib [96]. To assess the therapeutic property of [^131^I]PARPi, mice bearing U87-p53 tumor cells were assigned to three different cohorts: a control group (treated with PBS) and two fractionated treatment groups with either the therapeutic agent [^131^I]PARPi (3 × 14.8 MBq) or its non-radioactive version [^127^I]PARPi [96]. A median survival of 29 days was observed in the treatment group with [^131^I]PARPi while the control group and the group with the non-therapeutic [^127^I]PARPi had a lower median survival (22 and 20 days, respectively) (Figure 5A) [96]. The Auger-emitting version of the same compound, **[^123^I]MAPi**, showed a 16-fold greater cell killing potency compared to [^131^I]PARPi (EC_50_ = 69 nM and 1148 nM, respectively) and induced higher levels of DNA damage [97]. After intratumoral injection, the tracer was retained in tumors at high levels (40% ID/g 18 h p.i.), which could be blocked with a systemic olaparib pre-injection, and uptake in other organs remained low. Treatment of TS543 tumor-bearing animals with a single intratumoral dose of [^123^I]MAPi (0.37–1.11 MBq) led to an increase in survival (58 days vs. 40 days in the control group). These results were confirmed in a second cohort, where the radiotherapeutic was delivered via an osmotic delivery pump over a prolonged time. In this setup, survival increased from 48 days in the control group to 72 days in the treated group and the treatment was tolerated well by the animals [97]. Subsequently, an improved synthesis route for the ^123^I-labeling was developed using a single step ^123^I-iododestannylation reaction, yielding higher molar activity (A_m_) of 11.8 GBq/µmol compared to previous work with A_m_ = 3.9 GBq/µmol [98]. While intratumoral injection might be considered for glioblastoma treatment clinically, for other tumor entities, it is not an option. Therefore, the therapeutic potential of [^123^I]MAPi after systemic injection was evaluated in a colorectal cancer model comparing p53^+/+^ to p53^−/−^ models [99]. In tumors, PARP1 expression is elevated in the nucleus, increasing the likelihood for the Auger-emitting isotope to induce DNA damage. The combination of high PARP1 expression and genomic instability in tumors, e.g., via a p53 loss, could explain the promising therapeutic efficacy and tolerable toxicity of [^123^I]MAPi after systemic application, which needs to be confirmed in further studies.

**Table 3:** Overview of in vivo efficacy studies using PARP-targeted radiotherapeutics.

Agent	Publication	Tumor Model	Mouse Strain	Treatment Groups	Median Survival
[^211^At]MM4	[100]	GL26 (syngeneic) (Glioblastoma)	CB57BL/6J	(1) Control (2) 1 × 36 MBq/kg (~720 kBq) i.v. (3) 3 × 200 µg PD-L1 i.p. (4) Combination	PFI *: (1) 1 day (2) 21 days (3) 38 days (4) 65 days
	[101]	IMR-05 (Neuroblastoma)	SCID Hairless Congenic	(1) Control (2) 1 × 555 kBq i.v. (3) 1 × 1110 kBq i.v. (4) 1 × 1480 kBq i.v. (5) Fractionated (370 kBq i.v. 2 × weekly)	(1) 35 days (2) 61 days (3) 65 days (4) 10 days (toxicity) (5) 80 days
[^123^I]MAPi	[96]	U87-p53/tdTomato-CBRluc-Neo (Glioblastoma)	CrTac:NCr-Fo	(1) Vehicle PBS i.t. (2) 1 × 9.9 nmol [^127^I]PARPi i.t. (3) 3 × 14.8 MBq [^131^I]PARPi i.t.	(1) 22 days (2) 20 days (3) 29 days
	[97]	TS543 (Glioblastoma)	CrTac:NCr-Fo	(1) Vehicle i.t. (2) 1 × 0.37–1.11 MBq [^123^I]MAPi i.t. (3) Vehicle osmotic pump delivery (4) [^123^I]MAPi osmotic pump delivery	(1) 40 days (2) 58 days (3) 48 days (4) 72 days
	[99]	HCT116 p53^+/+^	CrTac:NCr-Fo	(1) Vehicle (2) 5 × 74 MBq [^123^I]MAPi i.v.	(1) 3.429 weeks (2) 3.286 weeks
	[99]	HCT116 p53^−/−^ (Colorectal cancer)	CrTac:NCr-Fo	(1) Vehicle i.v. (2) 5 × 80 µg/kg [^127^I]MAPi i.v. (3) 5 × 74 MBq [^123^I]MAPi i.v.	(1) 2.429 weeks (2) 3.071 weeks (3) 3.714 weeks

* PFI: Progression free interval.

For therapy, mice were administered 5 cycles of up to 74 MBq [^123^I]MAPi, which led to an increase in median survival in the [^123^I]MAPi treated group in HCT116 p53^−/−^ animals (3.2 weeks) compared to the vehicle treated controls (2.4 weeks) (Figure 5B), but not in HCT115 p53^+/+^ animals ([^123^I]MAPi: 3.3 weeks, vehicle: 3.4 weeks), supporting that loss of the tumor suppressor p53 lead to increased sensitivity. Although biodistribution data showed that large fractions of [^123^I]MAPi pass through the hepatobiliary system and uptake in several organs was higher than in tumors, only minimal systemic toxicity was observed in a toxicity study after GMP guidelines. It is hypothesized that during excretion, metabolism confines the agent to the perinuclear region of the cell and therefore puts it outside the range of an Auger-emitter to achieve significant damage upon cellular DNA [99]. 

Recently, another therapeutic study of an Auger-emitting PARPi, **[^125^I]PARPi-01** (isotopologue of [^131^I]I2-PARPi from [57]), was published (Morgenroth Lab) [102]. To assess the theranostic efficacy of the Auger electron emitter on triple negative breast cancer (TNBC), the tracer was evaluated in 11 different TNBC tumor cell lines, including BRCA-mutated and BRCA-wt cell lines. Specifically, [^125^I]PARPi-01 uptake was shown via olaparib blocking in MDA-MB-231 cells [102]. 

While some cell lines already showed a sensitive response to [^125^I]PARPi-01 monotherapy at lower concentrations than olaparib, the therapeutic response could be improved in non-responsive cell lines using a combinatorial treatment of [^125^I]PARPi-01 with the chemotherapeutic drug Dox-NP. The observed responses were consistent across a panel of assays including cell cycle analysis, apoptosis quantification, and colony formation assays [102]. If these results could be confirmed in vivo, [^125^I]PARPi-01 could be another interesting candidate for PARP radiotherapy.

### 5.2. Rucaparib-like Radiotherapeutics

The lab of Robert Mach developed an ^125^I-labelled PARP1-targeted tracer, based on the same scaffold as [^18^F]FTT [103]. **[^125^I]KX1** showed uptake in HCC1937 and MDA-MB-231 xenograft models, where a tumor uptake of 5% ID/g 2 h p.i. (HCC1937) and 3% ID/g 2 h p.i. (MDA-MB-231) was observed [103]. These data aligned with the known PARP expression levels of these cell lines and were also confirmed by ex vivo autoradiography of the tumors of both cell lines. However, unlike [^18^F]FTT, tumor uptake could not be significantly blocked by pre-injection of olaparib [103]. Further, [^125/123^I]KX1 was tested in ovarian cancer cells and human ovarian cancer xenograft mouse models [104]. In vitro experiments showed PARP1-dependence of the cell killing effect and a dose-dependent increase in the number of γH2AX foci after treatment with [^125^I]KX1 [104]. The authors also showed a dose-depending increase of apoptosis on tumor slices from patients upon [^125^I]KX1 treatment [104]. [^125^I]KX1 was evaluated for treatment in neuroblastoma models where its cytotoxicity was 10^4^–10^6^ times higher than its non-radioactive precursor KX1 across a panel of 19 cell lines [87]. In this study, an α-emitting KX1 version, **[^211^At]MM4**, was presented and showed significantly higher cell-killing potential than [^125^I]KX1, indicating that much lower doses would be needed to induce therapeutic effects. In vivo tumor dosimetry confirmed the superior therapeutic properties of [^211^At]MM4 over [^125^I]KX1, yielding a 150-times higher tumor nuclei dose per decay (radiation dose of ~ 35 cGy/decay vs. 0.1 cGy/decay, respectively) (Figure 5C) [87]. Hence, [^125^I]KX1 would require significantly higher activity than [^211^At]MM4 for equivalent in vivo efficacy. In combination with the long half-life of ^125^I, this could limit its in vivo potential. Moreover, immunohistochemistry confirmed that [^211^At]MM4 caused dose-dependent DNA damage among neuroblastoma cell lines, resulting in an increase of ƴH2AX foci [101]. Additionally, comparing the sensitivity of UWB1.289 (BRCA1 deficient) and UWB1.289-BRCA1 restored cells towards [^211^At]MM4, no difference was found, suggesting the therapeutic effect of ^211^At does not depend on the HR status of the cell line [101]. Lastly, a therapy study in IMR-05 tumor-bearing mice showed significantly increased median survival in the treatment groups (555 kBq and 1110 kBq single dose of intravenous [^211^At]MM4) over the control group (61 and 65 days vs. 35 days, respectively) [101]. In addition, the animals tolerated the treatment well and showed no weight loss or other signs of systemic toxicity, rendering [^211^At]MM4 a promising candidate for further evaluation as radiotherapeutic PARPi. 

In addition to inhibiting PARP, it is also feasible to inhibit other tumor escape pathways. One such method is the blocking of the PD-1 immune-checkpoint, which is normally used by the tumor cells to evade the tumor surveillance mechanism of the body [100]. In order to enhance the immune-checkpoint blockade, the α-emitter [^211^At]MM4 was tested in mono- and combination therapy on mice bearing GL26 glioblastoma tumor cells (Figure 5D) [100]. Hereby, average tumor response was the greatest (100%) for the combination treatment compared to the mono treatments with either 200 µg anti-PD-1 (83.6%) or 36 MBq/kg [^211^At]MM4 (58.2 %) [100]. Similar results were observed for the average progression free intervals (65, 36.4 and 23.2 days, respectively) and for the percentages of disease-free mice at the end of the study (100%, 60% and 0%, respectively), suggesting [^211^At]MM4 to be a potential candidate for combinatorial therapy of glioblastoma with PD-1 immune-checkpoint blockade [100].

A second ^125^I-labelled rucaparib analogue was reported, namely **[^125^I]KX-02-019** [105]. This compound is a modified version of [^125^I]KX1 that features a bicyclical benzimidazole. Its K_i_ value was in favorable range (13.9 nM) and biodistribution studies with mice bearing EMT-6 tumors showed tumor uptake of 1% ID/g at 2 h p.i. [105]. Although this was lower than other PARP imaging agents, tumor-to-muscle ratios were about five and partial deiodination in vivo was found due to thyroid uptake. Surprisingly, this study revealed higher affinity of the radiotracer towards PARP2, and therefore may be useful to predict treatment response to PARPi therapy more precisely [105].

To briefly compare the results observed with both therapeutic isotopes ^125^I and ^211^At, it can be stated that, although based on the microdosimetry of the nuclides, ^125^I should be more effective than ^211^At at cell killing when bound or in very close proximity to the DNA; PARP-targeted ^211^At therapy was much more potent than ^125^I therapy. 

## 6. Considerations for Clinical Manufacturing 

Of the many radiolabeled PARP imaging and therapeutic agents, only two are currently on clinical trials: [^18^F]PARPi and [^18^F]FTT. Next to the general suitability of a PARP-targeted tracer for translational/clinical imaging, several factors can hinder the progression of radiopharmaceuticals from the preclinical to the clinical phase and large scale clinical routine production, including choice of chemicals, feasibility of automation, total synthesis time, and final dose achievable [106,107]. In the following, we will outline challenges for PARP imaging agents related to synthesis automation, upscaling, and materials suitable for human use.

**Automation**. The vast majority of radiopharmaceuticals with clinical applications are produced on an automated synthesis platform. Exceptions can be found, most commonly among compounds labelled with radiometals. These radiochemicals typically require fewer steps and no purification, making manual synthesis possible without exposing the operator to high levels of radiation. However, in the vast majority of cases, and especially with ^18^F and ^11^C, radiosynthesis automation is a fundamental step towards the clinical validation of a radiopharmaceutical. Full automation allows radiopharmacies to start the synthesis at a much higher radiation levels than manual processes, as the operators set up the equipment before delivering the activity in the hot cell and are therefore protected from radiation exposure. Additionally, a fully automated system ensures synthesis reproducibility, with parameters, including pressure, time, and temperature, being finely controlled. Finally, the most likely source of microbiological contamination in a GMP laboratory is a human operator; thus, limiting manual intervention reduces the risk of contaminating the final product [108]. For some of the PARP radiopharmaceuticals discussed in this review, namely the BODIPY analogue of [^18^F]PARPi, [^18^F]PARPi-FL, the ^18^F-fluoroethyl analogue of AZD2461, the ^18^F-fluorethyl version of rucaparib, [^18^F]SuPAR, [^18^F]olaparib, and [^18^F]AZD2461, an automated procedure was already published [34,52,55,56,66,74]. For many, however, only a manual radiosynthesis was performed, and in some cases the reported procedures might be difficult to perform on an automated platform. Implementation on cassette-based systems, for example, need to consider limitations caused by dead volumes; therefore, processes that use very small volumes will likely need to undergo a series of additional tests to adjust. Furthermore, development of automated processes for particularly long and convoluted manual syntheses might prove challenging because of hardware limitations. Most systems have a limited number of positions available; therefore, processes requiring extra steps, such as multiple filtrations or solid phase extractions, will struggle to be accommodated on a smaller system [71,72,77]. Few systems have more than one heating reactors and/or magnetic stirrers; therefore, processes requiring these additional components will likely need re-examination, depending on the equipment available on site [72]. Typically, only one column can be connected to an automation system to perform the purification step; purifications requiring more than one column would most likely need to be revised [77]. Finally, processes requiring some sort of solid support, which must be removed at a subsequent step, will similarly need revision for successful implementation on an automated system. The synthesis of [^18^F]AZD2281, for example, requires magnetic removal of the beads used for the labeling [32,58]. 

**Scale-up**. As operators have to intervene heavily during the synthesis, manual and semi-automated radiosynthesis procedures are mostly carried out starting with smaller radiation levels than the amounts typically used for clinical applications.

Radiosynthesis scale-up, especially of radiopharmaceuticals made with short-lived isotopes, has several benefits, including increased final dose, and therefore is able to scan more than one patient with a single synthesis and transfer of doses to centers at certain distances from the production site. For instance, while the starting activity of manually synthesized ^18^F PET tracers herein reviewed was reported, it was, in most cases, in the 500–1800 MBq range, and it could be increased to 20–30 GBq for some automated syntheses [55,56]. However, there are also certain challenges involved in scaling-up radiosynthesis. Decomposition of the reformulated product due to radiolysis, which is more likely at high starting activity, is the major concern; this occurrence is caused by the formation of highly reactive species (hydroxyl radicals, aqueous electron, and superoxide) from water, and can be mitigated by adding anti-oxidants; for instance, radiolysis of [^68^Ga]-NOTA-sdAb was prevented for up to 3 hours with the combination of 20% ethanol and 5 mg ascorbic acid [109]. Occurrence of radiolysis of the final product experienced during the scale-up phase have been previously reported. The radiochemical purity of [^18^F]AV-19, a PET tracer for amyloid plaques, decreased to 73% when the synthesis was scaled up to 66.6 GBq of ^18^F due to the decomposition of the product into four polar radioactive species [110]. Radiolysis issues were not considered in the PARP imaging literature, presumably because no large-scale production was reported to date. Small-scale radiosynthesis methods have been improving, including droplet radiochemistry and microfluidics, and show many advantages, such as lower costs and shorter processes; however, they are more suitable for preclinical use, since only small amounts of radiopharmaceuticals are produced [111]. Therefore, PARP-targeted PET tracers currently at the preclinical evaluation stage will likely need to undergo scaling-up, ensuring that the radiochemical yield is not negatively affected, and that the product is not subject to radiolysis, before moving to clinical tests.

**Materials**. Pharmaceuticals for human use must be declared safe for the aforementioned purpose; one of the requirements towards this goal is to prove that any impurity in the final formulation is within the permitted daily exposure (PDE) for the specific administration route. The ICH provides guidelines on the PDE values of various types of impurities [112]. Of particular interest are byproducts, residual solvents, and elemental impurities. While radiopharmaceuticals usually go through several steps, such as solid phase extraction and column purification, that could remove these unwanted components, proving that the PDE values are within the limits is still necessary.

Solvents are classified by the ICH guidelines into four groups. Class I solvents are to be avoided, either because they are known or suspected human carcinogens, or because of environmental concerns. Class II solvents should be limited in pharmaceutical products because of their inherent toxicity. Class III solvents have low toxic potential. Class IV includes solvents for which no adequate toxicological data was found. Most radiolabeled PARP agents in this review are synthesized in solvents already classified by the ICH guidelines, and for some of which the PDE is reported; notable examples are: (i) acetonitrile (class II, PDE = 4.1 mg/day) [32,68,71], occasionally mixed with other solvents (*tert*-butyl alcohol, class II, PDE = 35 mg/day [58], used for [^123^I]I-PARPi), or methanol, class II, PDE = 30 mg/day [97], used for [^123^I]MAPi); (ii) *N*,*N*-dimethylformamide (class II, PDE = 8.8 mg/day), used for [^18^F]FTT [66] and (iii) dimethyl sulfoxide (class III, PDE = 50 mg/day), used, among others, for [^18^F]SuPAR and [^18^F]PARPi [42,55,74,96]. Some, however, are synthesized in solvents that are not included in any of the four groups in the ICH guidelines, and do not have readily available toxicological data. For instance, [^18^F]olaparib, [^18^F]AZD2461, and [^18^F]rucaparib are synthesized in 1,3-dimethyl-2-imidazolidinone. In such cases, the clinical validation radiochemist will likely need to re-optimize the radiosynthesis in a different solvent [52,56,72]. Additionally, inclusion in the ICH guidelines does not imply that a standard test is described in a Pharmacopoeia; in such cases, in-house methods must be developed. Innovative radiosynthetic approaches have vastly increased the number and nature of molecules that can be potentially radiolabeled. However, these methods frequently require metal catalysts and mediators; most commonly, copper compounds are used in both click chemistry and boronic esters labeling [52,56,72,74]. Copper, along with other commonly used metals such as tin, chromium, and lithium, is classified as a class III element according to ICH guidelines, i.e., considered to have relatively lower toxicity by oral route of administration, but requires risk assessment for parenteral and inhalation routes. While the PDEs of class III elements are relatively high (generally >500 µg/day, specifically 340 µg/day for copper via parenteral route), copper compounds have been frequently used in larger amounts, and therefore copper levels in the final dose must be monitored to ensure compliance. These aspects, which are key elements of the radiochemical production, are only some the points that must be taken into account during the development of a GMP-compliant radiopharmaceutical [106,107,113]. A full quality control system of the final product also needs to be in place, in order to determine radionuclidic, radiochemical, and chemical purity of the dose, in accordance to pharmacopoeias standards. Furthermore, a system of quality assurance is necessary to monitor compliance with standard operating procedures, including batch release approved by a Qualified Person.

Taking all these points into account, while a vast selection of PARP-targeted tracers is a constructive contribution to this field, when clinical application is the aim, the intrinsic limitations of the validation process should be kept in mind also during the preclinical development stage.

## 7. Conclusions

PARP-targeted imaging and radiotherapy is a highly active and rapidly moving field that emerged almost in parallel to the first approvals for PARP inhibitors. Two PARP-targeted PET imaging agents, the olaparib-based [^18^F]PARPi and the rucaparib-based [^18^F]FTT, have entered clinical evaluation and more, e.g., [^18^F]olaparib, are likely to follow. These early clinical studies indicate safety and feasibility of visualizing tumors/quantifying PARP1 expression in a range of tumor types. However, due to the nature of phase 0 and 1 studies, they only include small patient cohorts, and therefore cannot provide conclusive data on their potential to improve clinical standard-of-care yet. Two clinical applications of interest are the selection and monitoring of patients for PARPi therapy (as companion diagnostic) and imaging of tumors that cannot be reliably imaged with existing tracers, such as [^18^F]FDG. However, additional applications could emerge, including imaging-based response prediction to PARPi or risk stratification of patients. It can be hoped that PARP imaging agents will advance to phase II and III studies based on the encouraging results of early phase studies, to establish their value for specific clinical applications. In addition, we reported on exciting developments in the field of PARP-targeted radiotherapy. Several groups could show promising preclinical anti-tumor efficacy of α- and Auger-emitting agents as monotherapy or in combination with immune checkpoint inhibition. At first look, these agents display moderate tumor uptake compared to considerable uptake in non-target organs (e.g., high PARP1 expressing organs such as the spleen), but toxicity studies showed tolerable safety profiles at efficacious doses in mice. Since PARPi are cell permeable and bind to the PARP1 enzyme once it is bound to damaged DNA in the nucleus, alpha and Auger-emitters decay in close vicinity to the DNA, where they cause lethal damage. The higher PARP1 expression of tumor cell nuclei is a possible explanation for the selective toxicity in tumor cells, but further mechanistic studies are necessary to confirm this or investigate other explanations. Another possible reason is that a certain genetic makeup of tumors is necessary for a high sensitivity to PARP-targeted radiotherapy. Interestingly, while some studies report increased sensitivity in the presence of HR or p53 mutations, other studies report independence of sensitivity from such factors. Overall, more data and improved PARPi-targeted radiopharmaceuticals are eagerly awaited to gain a better understanding of the potential clinical impact of PARP-targeted imaging and therapy. 

## Figures and Tables

**Table 2:** Overview of radioisotopes used in PARP-targeted therapy [86,93,94,95].

	Isotopes			
	^123^I	^125^I	^131^I	^211^At
**Half-life**	13.2 h	59.3 d	8.0 d	7.2 h
**Major decay mode**	EC	EC	ß^−^ decay	α decay
**SPECT** **imaging** (abundance)	ƴ: 159 keV (abundance: 83%)	ƴ: 35.5 keV (abundance: 7%)	ƴ: 364.5 keV (abundance: 82%) (high energy coll.)	K x-rays (77–92 keV)
**ß^−^ energy**	none	none	606 keV (abundance: 90%)	none
**α energy**	none	none	none	1 α/decay (5.9 MeV–7.5 MeV)
**Auger energy**	11 AE/decay	21 AE/decay	none	6.3 AE/decay

EC: electron capture; AE: Auger electrons.
